# Cellulose-Based Aerogels for Environmentally Sustainable Applications: A Review of the Production, Modification, and Sorption of Environmental Contaminants

**DOI:** 10.3390/polym17020236

**Published:** 2025-01-18

**Authors:** Fernanda Wickboldt Stark, Pascal Silas Thue, André Luiz Missio, Fernando Machado Machado, Rafael de Avila Delucis, Robson Andreazza

**Affiliations:** 1 Environmental Sciences Postgraduate Program, Center of Engineering, Federal University of Pelotas, R. Benjamin Constant 989, Pelotas 96010-020, RS, Brazil; fernandastark@yahoo.com.br (F.W.S.); pascal.thue@ufpel.edu.br (P.S.T.); andreluizmissio@gmail.com (A.L.M.); fernando.machado@hotmail.com.br (F.M.M.); robsonandreazza@yahoo.com.br (R.A.); 2 Graduate Program in Materials Science and Engineering (PPGCEM), Technological Development Center, Federal University of Pelotas (UFPel), Pelotas 96010-610, RS, Brazil

**Keywords:** sustainable products, environmental contamination, environmental recovery, sustainable aerogels

## Abstract

Environmental pollution, stemming from the disposal of contaminants, poses severe threats to ecosystems and human health. The emergence of a new class of pollutants, termed emerging contaminants (ECs), in soil, water, and air has raised global concerns, aligning with the UN 2030 Agenda’s Sustainable Development Goals. Aerogels, three-dimensional structures with high porosity and low density, offer promise in addressing this issue. Cellulose-based aerogels, derived from abundant, renewable, and biodegradable sources, particularly stand out for their potential in adsorption applications. However, challenges arise in water and wastewater treatment due to cellulose aerogel’s inherent hydrophilicity. To overcome this limitation, incorporating new components and employing modification processes becomes essential. This article explores the production phases and diverse modifications of cellulose aerogels, aiming to enhance their adsorption capabilities for various environmental contaminants. By addressing hydrophilicity issues and developing stable composites, cellulose aerogels can contribute significantly to efficient and sustainable solutions in the quest for cleaner ecosystems and improved human health.

## 1. Introduction

Environmental pollution can be described as introducing dangerous substances into the environment, often derived from polluting or contaminating substances. Some widely known contaminants are plastics, particles, sulfur, nitrogen and carbon oxides, volatile organic compounds, and toxic elements such as heavy metals [1]. Furthermore, a new class of contaminants is being detected in water, soil, and air through more sensitive analytical techniques [2]. These contaminants are called emerging contaminants (ECs) and cover a wide variety of chemical substances; among these are pesticides, pharmaceuticals, personal care products, hormones, sunscreens, illicit drugs, perfluorinated compounds, disinfection by-products, nanomaterials, and microplastics [3,4].

The inadequate disposal of these environmental contaminants is a problem for living beings as it affects biodiversity by contaminating the physical and biological components of the terrestrial and atmospheric systems. Environmental living organisms are constantly affected by pollutants through direct contact or the bioaccumulation of chemicals. Toxic chemicals also put human health at risk through contact with contaminated soil, water, air, and food products [5].

Removing and treating these contaminants is a challenge that must be achieved to avoid more significant problems for future generations and improve environmental quality. In 2015, the United Nations (UN) General Assembly approved 17 Sustainable Development Goals (SDGs) that cover issues ranging from human health to the protection of aquatic life [6,7]. The removal and treatment of environmental contaminants in water and wastewater are directly aligned with several SDG objectives, as exemplified by SDG 6, which aims to “ensure the availability and sustainable management of water and sanitation for all”.

Given this, various technologies are being developed to remove contaminants, including physical, chemical, and biological methods, or combining two or more treatment techniques [8]. The adsorption process has been recognized as one of the most promising techniques for removing organic contaminants in solution [9,10,11]. This is due to its low cost, ease of operation, absence of sludge generation, and absence of toxic intermediates [12,13].

Aerogels are three-dimensional solid materials composed of more than 90% air, resulting in a highly porous structure and, therefore, low density [14,15,16,17]. This porous material is obtained by replacing the liquid part of the sol-gel compound with air through a drying process [16]. Notable characteristics such as high surface area, high mesoporosity, low thermal conductivity, and modifiable surface chemistry make this material one of the most versatile for technical applications [15,18]. Aerogels are being used for biomedical applications [19], acoustics [15], thermal insulation [20], supercapacitors [21], and for the adsorption of contaminants to minimize environmental problems [22,23]. They can be obtained through mineral [24], synthetic [25], and natural [26] materials.

Recent research has been focused on the development of aerogels made from biopolymers. These biopolymers contain the characteristics of biodegradability, biocompatibility, non-toxicity, and easy accessibility [27]. Using biopolymers instead of petroleum-based polymers, which degrade slowly and are often associated with environmental pollution in the manufacture of aerogels, results in a more sustainable and ecologically friendly product [28]. Furthermore, using biomass as a raw material in manufacturing biopolymers represents a beneficial approach to improving waste management and contributing to the preservation of the environment [12].

Cellulose is known to be the most abundant, natural, renewable, and biodegradable polymer on earth [29]. It can be extracted from various sources, such as wood, annual plants, microbes, and animals [30]. Cellulose is a straight-chain polysaccharide with a repeating structure of β-(1,4)-D-glycosidic units. These units establish intramolecular solid hydrogen bonds [31], giving cells fibrillated structures. Nanocellulose is extracted from cellulose microfibrils [32]. Nanocellulose is a light and resistant material that consists of fibrils (CNF) and cellulose crystals (CNC) on a nanometric scale. This innovative material exhibits pseudoplastic behavior and can form gels or liquids of viscous consistency under usual conditions [33].

Nanocellulose-based aerogels have stood out as leaders in research on the adsorption of contaminants, as they have consistently demonstrated superior adsorption capacity when compared to other widely used adsorbents, such as activated carbon, silica, zeolite, and chitosan [29]. Due to its network structure, it presents advantageous characteristics, such as a large specific surface area and low density [34]. Furthermore, another favorable aspect of this material is the abundance of hydroxyl functional groups, which can provide active sites for the adsorption of contaminants or even enable modifications through bonds with other chemical compounds [35].

Despite their promising characteristics, authors have found challenges in the applications of nanocellulose aerogels due to their hydrophilicity characteristics, lack of specific functional groups, and insufficient mechanical properties for the adsorption of contaminants [26,35,36]. As a result, chemical and physical modifications have been studied to alter these characteristics and improve the adsorption capacity.

Thus, this study aims to conduct a literature review on the manufacture of nitrocellulose-based aerogels and detail the modifications implemented to improve their physicochemical and mechanical properties, focusing on the sorption of contaminants. This research is essential to clarify the aerogel production process, as most reviews and research focus on the adsorption process of contaminants in aerogels. Furthermore, we seek to encourage more significant investment in research related to nanocellulose aerogels, aiming for their eventual application on a real scale.

## 2. Production of Nanocellulose Aerogel

**Figure 1:**
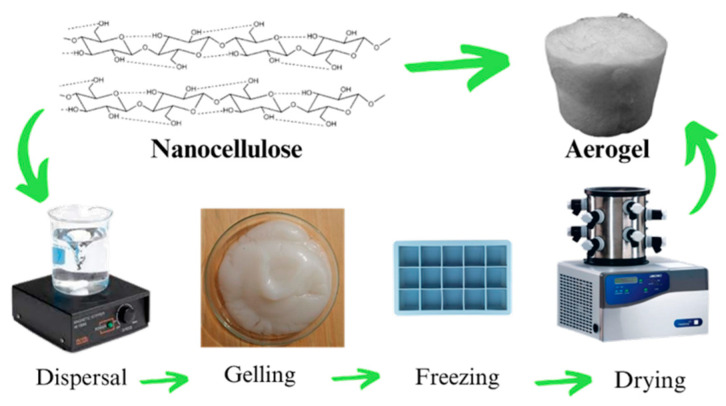
Simplified process for preparing nanocellulose aerogel by freeze-drying. Adapted from [38,39].

The first successful production of aerogel occurred in 1931 by Kistler. Typically, nanocellulose aerogels are produced in three steps: nanocellulose dispersion, nanocellulose gel formation, and gel drying [22]. Initially, (1) nanocellulose is dispersed, then (2) nanocellulose gel is formed, which is also known as sol-gel state. Finally, (3) nanocellulose aerogels with a three-dimensional porous structure are dried through freeze-drying, although supercritical drying using CO_2_ is also used to produce aerogels, with a major limitation of this process being its high cost [37]. Furthermore, the fabrication of nanocellulose aerogel encompasses significant additional steps, including determining the concentration of nanocellulose and other components, the freezing procedure, and modifications, which will also be covered in this article Figure 1.

### 2.1. Nanocellulose Concentration

**Figure 2:**
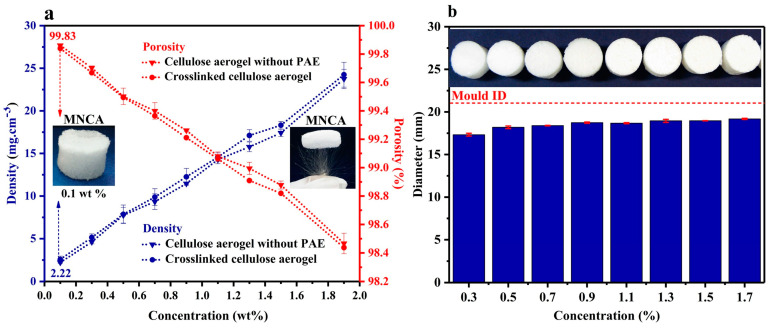
Changes in density and porosity (**a**), and diameter (**b**) through different cellulose concentrations [43]; Copyright 2021, reproduced with permission from Elsevier.

The cellulose concentration for preparing an aerogel depends on the specific objectives of the experiment or the desired application. Due to the high number of parameters to be considered in the preparation of cellulose aerogels, making generalizations about specific properties and their impacts on the adsorption of contaminants is a challenging task. The concentration of nanocellulose can affect several properties of the aerogel, such as its porosity, density, mechanical resistance, shrinkage, and sorption capacity (Figure 2). Porosity, pore size, and shape can be controlled by changing the concentration of nanocellulose material [40] (e.g., increases in nanocellulose concentration evidently cause increases in the density of aerogels, in addition to changes in the specific surface area [41]). Furthermore, increases in cellulose concentration accompany increases in specific surface area due to the progressive fractionation of pores into new and smaller pores and are not necessarily linked to increases in the thickness of the pore walls [42].

Zhang et al. [44] prepared aerogels with various concentrations (1, 2, and 3 wt%) of cellulose nanofibrils grafted with beta-cyclodextrin (CNF-CD). It was found that the apparent density, the capacity of water absorption, and the adsorption capacity of phenolic pollutants increased proportionally with increasing concentration of the CNF-CD solution.

In another study, Zhang et al. [45] produced an aerogel made from nanocellulose and sodium dodecyl sulfate. In order to obtain the ideal concentration of nanocellulose, aerogels were manufactured with different concentrations of cotton nanocellulose (0.2 to 1 wt%). A 3D structure was formed at all concentrations. However, the aerogel with 0.4 wt% nanocellulose showed a much larger porous structure than the others. Furthermore, with the increase in cellulose concentration (0.6 to 1% *w*/*w*), the polymers adhere to each other, which is not beneficial for forming the porous structure. The aerogel becomes fragile when the concentration is very low (0.2% *w*/*w*). Subsequently, the author also conducted tests to determine the best concentration of sodium dodecyl sulfate (0.1 to 0.3% *w*/*w*). The authors found that the ideal concentration was 0.2% *w*/*w*; below that, the foam formed is not as stable, and above that, it impairs the dispersion of nanocellulose.

The cellulose concentration can vary concerning the crosslinking and coupling agents used for the formation of the 3D structure of cellulose aerogel. While Udoetok et al. [46] employed epichlorohydrin (EPI), a resin, Zhang et al. [45] chose sodium dodecyl sulfate, a surfactant. Therefore, it is crucial to conduct tests with different cellulose concentrations and crosslinking agents to determine the optimal concentration, i.e., the one that provides the best structure and adsorption performance.

### 2.2. Dispersion of Nanocellulose and Aerogel-Forming Characteristics

For reproducible research results and for all nanocellulose products to meet quality criteria, stable dispersion is necessary [29]. Dispersion is the procedure by which solid nanocellulose particles are properly distributed evenly in a liquid, usually distilled or deionized water. During this process, particles are fragmented into smaller dimensions and spread homogeneously in the medium. Nanocellulose dispersion can be carried out using magnetic or mechanical stirrers, ultrasonicators, and homogenizers.

The dispersibility of nanocellulose is good in polar solvents. For example, nanocellulose has excellent dispersibility in water due to the strong interaction between hydroxyl groups and water molecules. In hydrophobic solvents, the dispersion of nanocellulose is impaired as it is a hydrophilic material. To overcome this, the surface chemistry of nanocellulose must be adjusted [47]. Zhu et al. [36] prepared aerogels with an initial suspension (1 wt%) containing two types of nanocellulose (cellulose nanofibrils—CNF, and pre-prepared cationic cellulose nanocrystals—CCNC) subjected to magnetic stirring for 1 h. In order to improve electrostatic adsorption, a solution of anionic sodium alginate (30% *w*/*w* relative to the total of CNF and CCNC) was slowly added to the suspension under magnetic stirring for 3 h to achieve a stable dispersion. Without the help of the anionic sodium alginate solution, the direct composition between the CNF and CCNC suspensions would lead to flocculation and sedimentation of the nanoparticles.

Other important characteristics in the formation of aerogels are porosity and specific surface area. The pores of aerogels are replicas of the ice crystals that appear during freezing. In this way, changing the size of ice crystals causes changes in the size and volume of pores present in cellulose-based aerogels. Because of this, the concentration of cellulose, the length of its fibers, the presence of impurities (such as lignin), and the intertwining density of the cellulosic fibers are significant attributes of the characteristics related to porosity. Fan et al. [48] studied the adsorption performance of aerogels based on carboxyl cellulose reinforced by montmorillonite (MTT) and functionalized with polyethyleneimine (PEI) through an amidation reaction. In their work, the authors detected that the increase in PEI content led to higher levels of intertwining between PEI and cellulose, restricting the growth of ice crystals and, after drying the aerogel, generating smaller pores and larger irregularly shaped pore channels. In contrast, Dilamian e Noroozi [43] attested that, by increasing the fiber content in their cellulose-based aerogel functionalized by polyamideamine-epichlorohydrin (PAE) resin, the percentage porosity was reduced due to pore shrinkage linked to chemical cross-slinking reactions of curing assisted by heat. Furthermore, the authors state that improvements in the aerogel adsorption process occurred because the silane-coated cellulose aerogel (s-MNCA) presented pores small enough to keep the adsorbed liquid within its porous structure.

### 2.3. Gelation and Crosslinking Process

**Figure 3:**
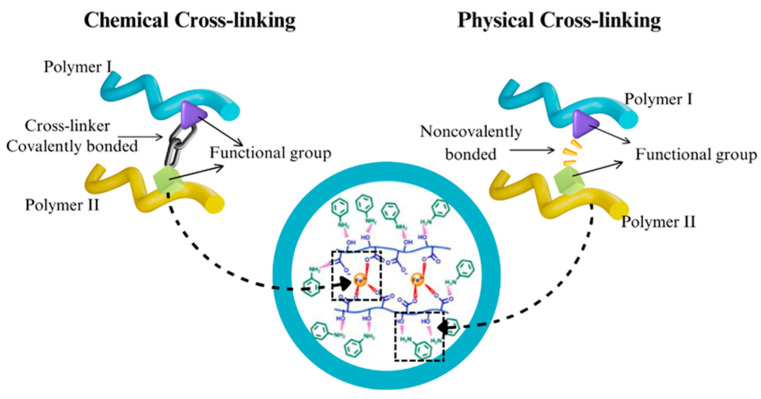
Difference between chemical crosslinking and physical crosslinking. Adapted from [49,53].

After complete dispersion of the aerogel components, the solution is subjected to a process that induces gelation, which can be achieved by adding crosslinking agents. During this process, nanocellulose molecules and other components organize themselves and form a three-dimensional gel-like structure. The gelation behavior of nanocellulose is commonly categorized into two main classes according to the nature of the gel: chemical crosslinking and physical crosslinking [22]. Natural polymers, mainly polysaccharides, are crosslinked by the reaction between hydroxyl or amino groups, forming water-insoluble crosslinked units [49] as shown in Figure 3.

Chemical crosslinking is established through irreversible covalent bonds between the reactive hydroxyl groups of nanocellulose [36] and other chemical reagents. Zhang et al. [44] and Maldonado et al. [50] used epichlorohydrin to crosslink beta-cyclodextrin into cellulose nanofibers. Mo et al. [35] introduced trimethylolpropane tris-(2-methyl-1-aziridine) propionate (TMPTAP) to form a specific covalent crosslinking network between cellulose nanofibril and graphene oxide. Camparotto et al. [51] conducted the coupling between cellulose nanocrystals and hydrophobic tannic acid using glutaraldehyde as a crosslinking agent. Glutaraldehyde is also used to crosslink chitosan, reacting with free amino groups to form imine bonds [49]. In a metal-organic framework (MOF) composed of zinc nitrate hexahydrate and cellulose, Cui et al. [52] used the crosslinking agent N, N′-methylenebisacrylamide (MBA).

Physical crosslinking involves weak intermolecular forces such as electrostatic interactions, hydrogen bonding, and hydrophobic interaction among polymer chains [49]. Lyu et al. [53] fabricated an aerogel from nanofibers of polyaniline and carboxylated cellulose nanofibers (CNFs) through a two-step method that provided improvements in mechanical strength for wastewater treatment. In this approach, FeCl3 and aniline were crosslinked in situ with carboxylated CNFs through the coordination interaction between Fe(III) and -COOH, and by physical crosslinking through hydrogen bonding between aniline and CNFs, respectively. Subsequently, in situ polymerization of PANI-NFs occurred in the CNFs hydrogel skeleton in an acidic environment. The aerogels exhibited the highest compression strength of 48.2 kPa at a compressive strain of 50% and superior adsorption performance of 600.7 mg/g for acid red G, anionic dye, and 1369.6 mg/g for methyl blue, cationic dye.

Mo and his team [54] also developed aerogels in two steps through physico–chemical crosslinking processes, using cellulose nanofibers with polyacrylamide. In the aerogel synthesis, initially, physical crosslinking occurred between the oxidized cellulose nanofibers by TEMPO (TOCNFs) and anionic polyacrylamide (APAM) through hydrogen bonding. Then, TOCNFs were chemically crosslinked in situ with APAM using the ring-opening reaction between the carboxyl groups of TOCNFs and the chains of APAM, along with trimethylopropane tris-(2-methyl-1-aziridine) propionate (TMPTAP). As a result, carboxyl and amino groups were introduced into the structure of the cellulose nanofiber, creating numerous active sites for the adsorption of heavy metal ions.

### 2.4. Aerogel Freezing

Pure nanocellulose does not form a thick gel without a chemical crosslinke. Therefore, freezing, followed by freeze-drying, is a highly suitable technique for its preparation [40]. Freeze molding is an economical, versatile, fast, and environmentally friendly method for creating 3D structures with well-defined macroporosity. The general idea is to combine the chemistry of materials with the physical principles of ice, producing porous, ultralight, and superelastic materials. The process is based on three essential steps: (1) directional freezing of the paste through contact with a cold surface, (2) the paste remains frozen for a controlled period, with the management of freezing parameters and directions, and 3) sublimation occurs of the ice crystals formed within the structure, which is reflected in the final porous structure. Furthermore, parameters such as temperature, cooling rate, pH, composition, and concentration of the sol-gel interfere with the direction and behavior of ice crystals [55]. Dilamian and Noroozi [43] produced highly porous (98.4–99.8%) and ultralight (density 2.2 to 24 mg/cm^3^) aerogels using the freezing method. The manufacturing involved freezing suspensions of rice husk cellulose nanofibers (MNCFs) by contact with liquid nitrogen and freezing in a freezer for 24 h. SEM micrographs showed a porous structure with various pore sizes, with the smallest arising from the nanofiber entanglement and the largest resulting from ice crystal sublimation during freeze-drying. Freezing with liquid nitrogen at −196 °C allowed the rapid formation of tiny ice crystals, concentrating the MNCFs.

### 2.5. Aerogel Drying: Supercritical and Freeze-Drying

To obtain the porous, low-density three-dimensional structure, the water or solvent present in the gel matrix must be removed in a controlled manner to preserve its structure. The most used techniques for drying aerogels are freeze-drying [34] and supercritical drying with CO_2_ [56].

In supercritical drying, the gel is exposed to high pressures and temperatures in an autoclave using a continuous flow of supercritical CO_2_. The CO_2_, with pressure and temperature above its critical point, is pressurized, acting as a substitute for the solvent in the gel matrix. After drying, CO_2_ is released through a controlled reduction in pressure and temperature, resulting in an aerogel with the same porous structure as the gel [57]. In a different way, freeze-drying is achieved by freezing the gel and then sublimating the ice crystals formed during the freezing process. This sublimation occurs in a vacuum chamber called a freeze dryer, in which the frozen gel transitions directly from the solid state to the gaseous state, forming porous aerogels [58].

Zhao et al. [59] synthesized microcrystalline cellulose aerogels using both drying methods. Their results indicated a superior performance of aerogels dried by supercritical drying with CO_2_ compared to freeze-dried aerogels. This is because supercritical drying with CO_2_ causes less damage to the sample structure. CO_2_, in its supercritical state, has an extremely low surface tension, which allows it to maintain the sample’s structure during the drying process. On the other hand, in the vacuum freeze-drying process, ice sublimation generates a significantly higher surface tension than CO_2_ in the supercritical state, resulting in pore collapse and different degrees of aerogel shrinkage. Generally, obtaining aerogels by freeze-drying results in more damaged structures, due to the irregularity that the ice crystals can present (which can subsequently harm the aerogel’s ability to withstand mechanical stress homogeneously). In addition, the disadvantages of the process of freeze-drying comprise the long processing time that the technique demands and the high consumption of electrical energy [60]

**Figure 4:**
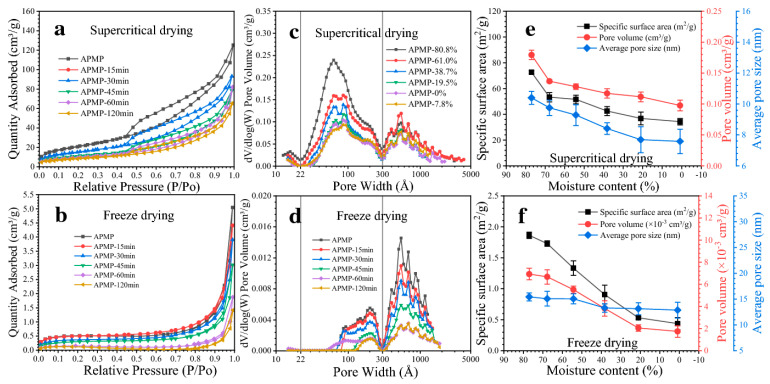
Nitrogen adsorption-desorption isotherms, pore size distribution, and pore parameters of (**a**,**c**,**e**) supercritically dried and (**b**,**d**,**f**) freeze-dried poplar alkaline peroxide mechanical pulp fibers with different moisture contents [61]; Copyright 2022, reproduced with permission from Springer Nature.

The morphological characteristics and porosity of aerogels vary significantly between freeze-drying and supercritical drying, as shown in Figure 4. The choice of drying technique is crucial to tailoring material performance for a specific application. If rapid dissolution in water is required, freeze-dried aerogels are ideal due to their irregular surface and large pores. On the other hand, dried supercritical aerogels are preferred to retain oil in the matrix structure, for they are more homogeneous, with fewer cracks and a smaller pore volume [58].

**Figure 5:**
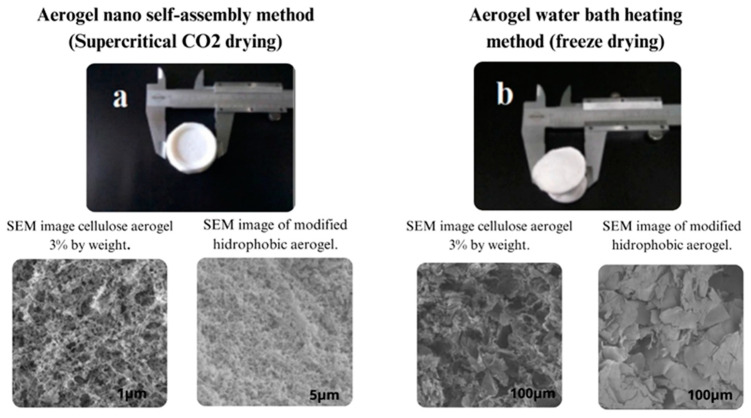
Comparison of SEM images of aerogels produced by supercritical drying processes with CO_2_ and freeze-drying. Adapted from [59].

Figure 5 compares SEM images of aerogels produced by supercritical drying processes with CO_2_ and freeze-drying. It shows the structural difference that the supercritical drying process can make, resulting in pores with a lower size structure and more irregularity than the freeze-drying process.

## 3. Modification of Nanocellulose Aerogel

Cellulose is rich in hydroxyl groups, as mentioned previously. Each cellulose unit has a primary hydroxyl group at C6 and two secondary hydroxyl groups at C2 and C3 [47]. The hydroxyl groups at C2 and C6 act as hydrogen bond donors for water. In contrast, the third group at C3 acts as a water-accepting hydrogen bond and donates hydrogen bonds to the oxygen atom of the intramolecular chain at C5 [62].

**Figure 6:**
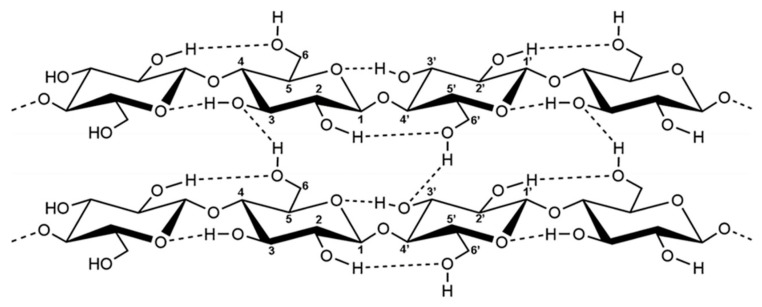
Molecular structure of cellulose [58].

Intramolecular and intermolecular hydrogen bonds between hydroxyls can quickly form physically crosslinked 3D gel networks (Figure 6). Furthermore, the active hydroxyl groups of nanocellulose allow combination and modification with specific functional fillers, leading to the development of diverse functional composite aerogels applicable in various environmental protection domains [63].

Notwithstanding, hydroxyl groups provide high hydrophilicity, which may be a problem for specific applications of nanocellulose aerogels [17], since this characteristic makes them absorb more moisture from the environment, causing damage to the network structure, and hinders their use in, for example, thermal insulation [64], flexible electronic devices [65], and catalysis in aqueous media [66]. To change the characteristics of nanocellulose aerogel, researchers have used different methods such as impregnation, coating, chemical vapor deposition, hydrothermal processes, and carbonization with different chemical processes such as silanization, esterification, and crosslinking.

### 3.1. Impregnation

The impregnation method consists of immersing or directly applying a particular substance to the nanocellulose aerogel, intending to allow the aerogel to absorb the substance. Chen et al. [17] modified cellulose nanofibril (CNF) aerogels with a facile two-step impregnation process. Firstly, the prepared pure CNF aerogels were impregnated in hydrolyzed silicon alcohols (methyltriethoxysilane) to make the silanol group react with hydroxyl groups to form a covalently bonded silane layer. They were then impregnated in an alkaline ammonia solution with pH 10 to make the silanol self-condense, forming polysiloxane particles. Impregnation by silanization modification made the CNF aerogels superhydrophobic (water contact angle up to 155.2°).

Doan and Chiang [67] developed a hybrid silica aerogel (SCA) with cellulose nanocrystals (CNC) in a single container and gelation at room temperature. Subsequently, the aerogel was impregnated with polyethyleneimine (PEI), resulting in SCA-PEI. The results indicated that CNC played a significant role in strengthening the gel structure, while silica improved its thermal stability compared to the original CNC materials. Impregnation with PEI considerably enhanced the CO_2_ capture performance (SCA-PEI—2.35 mmol/g at 70 °C with 50% PEI by weight), compared to SCA (0.25 mmol/g). This is due to the fact that SCA-PEI aerogel can adsorb CO_2_ through physical and chemical interactions between amino groups and CO_2_ molecules.

### 3.2. Coating

The aerogel coating process involves applying an external material layer to its surface, intending to give additional properties. The coating is also carried out by immersion (e.g., dip-coating), allowing the aerogel to absorb the material. Chhajed et al. [68] produced esterified aerogel through a two-step method: freeze-drying and dip-coating. The esterification process uses long-chain fatty acids, which form a covalent anchor on the cellulose surface. In this study, the aerogel was immersed in a solution with fatty acids for 1 h at a temperature between 60 °C and 70 °C. With increasing fat chain length, the surface wettability changed from hydrophilic to superhydrophobic, and the obtained aerogels were used for crude oil adsorption.

### 3.3. Chemical Vapor Deposition

Modification by chemical vapor deposition is a process that involves the controlled application of a thin layer of material onto the surface of the aerogel. The aerogel is placed in a vapor deposition chamber, where the coating material is evaporated from a source. This vapor condenses on the surface of the aerogel, interacting chemically, ensuring adhesion between the material and the aerogel. Rafieian et al. [69] developed a hydrophobic aerogel through vapor modification with hexadecyltrimethoxysilane (HDTMS) to absorb and remove oil and organic pollutants from water. The procedure consists of placing 3 mL of HDTMS in a 10 mL bottle and placing it in a 500 mL bottle together with the cellulose nanofibril aerogel, but without maintaining direct contact. The larger bottle is then sealed and placed in an oven at 155 °C for 1 h. Unreacted silanes are removed by drying the aerogel. The results revealed that, after modification, the aerogels exhibited a contact angle greater than 90° and were classified as hydrophobic materials. Tests conducted with two types of oils (kitchen and engine) showed that the oils were quickly and reversibly absorbed by the aerogels, with adsorption capacities of 78.8 and 162.4 g/g for engine oil and kitchen oil, respectively.

### 3.4. Hydrothermal Treatment

Hydrothermal treatment for aerogel modification is a process that involves the controlled exposure of the aerogel under specific temperature and pressure conditions in the presence of a solution containing the reagents or substances desired for aerogel modification. Liu et al. [70] produced an aerogel made of cellulose nanocrystals modified with acrylic acid to remove heavy metals using a hydrothermal treatment. The aerogel prepared with cellulose nanocrystals was placed in a 100 mL hydrothermal reactor and subjected to a hydrothermal reaction at 102 °C for up to 8 h in contact with 3 mL of acrylic acid. The adsorption tests with lead, cadmium, and copper showed excellent performance, with maximum adsorption capacities of 1.026, 898.9, and 872.4 mg/g, respectively.

Gu et al. [71] synthesized a nanocellulose aerogel for oil adsorption and organic solvents. To improve the hydrophobic property and adsorption capacity, nanocellulose, nanochitosan, and reduced graphene oxide were used to produce the nanocomposite aerogel through the hydrothermal method (150 °C for 1 h) combined with the freeze-drying method. The aerogel exhibited high hydrophobicity with a large water contact angle (115.26°) and high adsorption capacities of 171.85. ± 3.02, 159.64 ± 1.83, 153.22 ± 2.92, 149.60 ± 6.26, 139.93 ± 3.69, 132.47 ± 3.45, 176.82 ± 4.66, 128.70 ± 0.69, and 120.34 ± 5.57 mg/g for mineral oil, sesame oil, acetone, ethyl acetate, thiophene, pump oil, waste pump oil, kerosene, and ethyl alcohol, respectively.

### 3.5. Carbonization Treatment

Carbonization is a method that involves transforming aerogel into carbon through a controlled pyrolysis process. The interest in carbon aerogel production arises due to its specific applications, which are not feasible for unmodified cellulose aerogels (e.g., electromagnetic shielding [67], supercapacitors [68,69], and microwave absorption [70]). The advantage of producing carbon aerogels from nanocellulose structures lies in the significant potential for using different plant biomass and their unique porous structure and renewable characteristics, which highlights that nanocellulose aerogels are powerful substrates [71]. Additionally, carbon aerogels are widely used in adsorption separation processes due to their good chemical stability and high purity [72], without the high density of oxygenated functional groups present in the chemical structure of cellulose. In this process, the aerogel is impregnated with a carbon precursor. Next, the aerogel is subjected to heat treatment in a controlled environment. During pyrolysis, the carbon precursor decomposes, resulting in the formation of a carbonized structure.

Talari et al. [72] produced a carbon fiber aerogel derived from bacterial cellulose, produced by the bacteria Acetobacter-Xylinum from bacterial cultivation in a liquid medium. The production of aerogel was carried out through freezing and freeze-drying processes. The bacterial cellulose aerogel was pyrolyzed under an inert nitrogen atmosphere at 800 °C for 90 min to prepare the carbon fiber aerogel, with a 5 °C/min heating rate. The weight loss of the aerogel was around 55%. The results showed that the bacterial cellulose fibers were almost entirely converted into carbon. Adsorption tests were carried out with two contaminants, methylene blue and p-nitrophenol. The maximum adsorption capacities at room temperature and the original pH were 106 and 60 mg/g, respectively.

**Figure 7:**
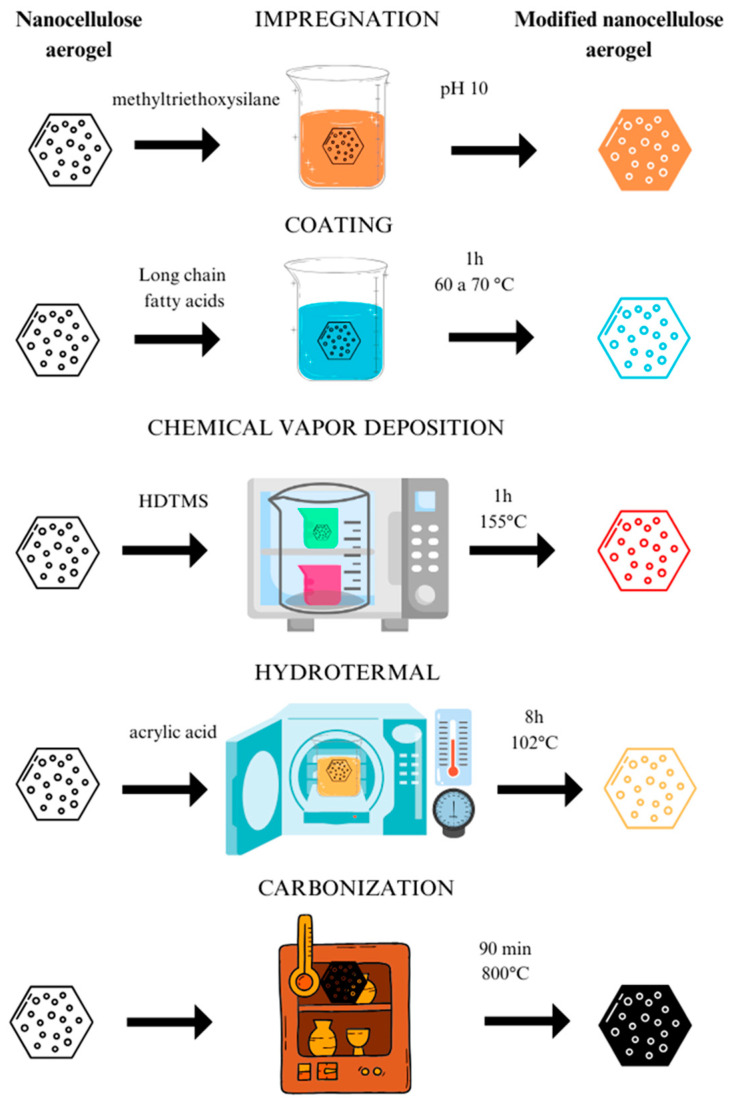
Summary of modification steps for nanocellulose aerogels.

Modifications of the nanocellulose aerogels can be done (Figure 7). Alatalo et al. [73] introduced nitrogen functionalities into aerogels produced from microcrystalline cellulose by hydrothermal carbonization. For this, cellulose was mixed with ovalbumin (from chicken egg white) and added to a reaction medium of water and sulfuric acid to catalyze the degradation of cellulose. The hydrothermal carbonization process was achieved by placing the mixture in a sealed glass inlet in a Teflon-lined autoclave and placed in a preheated oven at 180 °C for 5.5 h. After the reaction, the autoclave was cooled naturally, and the carbonaceous product was washed and lyophilized. After the process, a firm, stable aerogel with a dark brown color was obtained. The protein provided nitrogen functionalities (2.1 wt%) and was a structural directing agent, as these proteins form thermally induced aqueous gels when heated above the denaturation temperature. Adsorption tests on heavy metals such as chromium and lead demonstrated improved removal capacity of 68 mg/g and 240 mg/g, respectively.

## 4. Adsorption of Environmental Contaminants by Nanocellulose Aerogels

Adsorption is a phase transfer process that occurs at solid-gas or solid-liquid interfaces. It can also be defined as a process of adhesion of existing chemical species to a solid phase. In this context, the solid surface that provides the adsorption sites is called adsorbent, while the substance adsorbed on the solid surface is known as adsorbate [74]. The adsorption mechanism can be generalized in a few steps: (i) the adsorbate diffuses into the pores on the aerogel surface, (ii) the pores become filled and capillary forces restrict the adsorbate, which subsequently (iii) accumulates completely in the porous structure of the aerogel [43]. In this process, intramolecular and intermolecular interactions (e.g., van der Waals forces and hydrogen bonds) between the solid adsorbent and the adsorbate are essential factors for the adhesion of the compound [75]. Because of this, pore morphology and the efficiency of capillary forces are important topics that are linked to greater sorption capacity.

**Figure 8:**
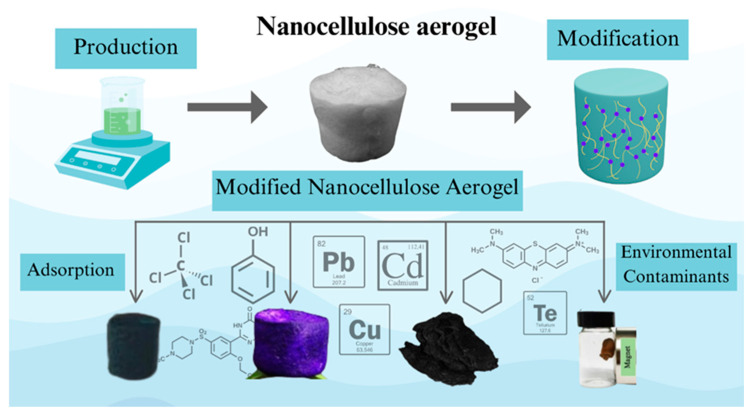
Production and application of modified nanocellulose aerogels in the adsorption of environmental contaminants. Adapted from [38,39,49,63,70].

Commercial adsorbents, such as graphene, activated carbon, and carbon nanotubes, are used to remove contaminants. However, they are expensive [76]. Therefore, many studies have been developed to produce low-cost and sustainable adsorbents. For example, the removal capacity of a natural clay adsorbent for acid red dye ranges from 421.9 to 1178.5 mg/g [11], while for reactive blue 19, it is 178 mg/g, and for reactive green 19 it is 361.1 mg/g [77]. Activated carbons demonstrate the ability to remove phenolic compounds in the range of 55.16 to 68.52 mg/g [78]. A graphene sponge exhibits a remarkable removal capacity of 1842 mg/g for nitrophenols [9]. The removal capacity of an emerging contaminant (omeprazole) by biochar has been recorded at 63.80 mg/g [79]. The studies shown in Table 1 indicate that the removal capacity of nanocellulose aerogels is comparable to that of low-cost adsorbents presented here. Figure 8 shows the production and application of nanocellulose aerogels in the adsorption of environmental contaminants.

### 4.1. Adsorption Parameters for Nanocellulose Aerogel

The effectiveness of the adsorption process is subject to several factors, including operating conditions (such as temperature, pH, and concentration level), properties of the adsorbate (such as chemical composition, polarity, molecular dimensions, and charge), and properties of the adsorbent (such as surface chemistry, residual surface charge, and textural parameters) [80].

The adsorbent dose plays a fundamental role in the adsorption process. In general, the adsorption of a solute increases with an increase in the concentration of an adsorbent because an increase in adsorbent concentration leads to an increase in active sites. However, the adsorption per unit weight of an adsorbent may decrease after increasing the concentration of the adsorbent due to interference caused by the interaction of active sites of an adsorbent [81]. Cui et al. [52] studied the impact of adsorbent dosage on adsorption efficiency using 0.5 to 5 g of adsorbent per liter. Increasing the dose from 0.5 to 3 g/L increased the removal efficiency from 38.52 ± 1.32 to 93.88 ± 2.52%, while the adsorption capacity decreased from 77.02 ± 2.63 to 31.29 ± 0.84 mg/g. Considering the economic cost and adsorption efficiency of the adsorbent, 2.0 g/L was selected by the authors as the ideal dose of adsorbent in a subsequent experiment.

According to Cui et al. [52], adsorbate concentration is also a crucial parameter to be considered when investigating adsorption, as concentration represents one of the main driving forces in the adsorption process. The authors employed concentrations ranging from 30 to 1000 mg/L of enrofloxacin. The adsorption capacity of ZIF-8 cattail cellulose aerogel (ZCCA) increased significantly with increasing enrofloxacin concentration at a constant temperature. When the concentration reached 700 mg/L, the active sites of the ZCCA aerogel were saturated, reaching equilibrium in adsorption capacity. This indicates that the increase in driving force, based on the concentration gradient, drove the transfer of pollutants from the solution to the surfactant sites of the ZCCA.

An affinity of aerogels with liquids and gases containing adsorbates plays a crucial role in the efficiency of adsorption processes. The high hydrophilicity of cellulose nanostructures promotes better electrostatic interactions with polar adsorbents, leading to numerous studies investigating the adsorption capacity of these aerogels with organic contaminants [82,83]. In this regard, the high hydrophilicity contributes to improvements in the adsorption capacity of polar products and antifouling properties [82], proving to be an important characteristic in water–oil separation systems and preventing the accumulation of organic matter [84].

pH is one of the most critical variables in the adsorption process, as it directly influences adsorption by adsorbents, impacting both the extent of adsorbate ionization and the characteristics of the adsorbent surface [81]. Liu et al. [70] conducted experiments with pH values from 1.5 to 5.5 in different solutions of 400 mg/L of Pb(II)(II), Cd(II)(II), and Cu(II)(II). The adsorption capacities of heavy metal ions gradually increased as the initial pH of 1.5 increased to 5.5. The influence of pH in the solution suggests that the carboxyl groups of acrylic acid grafted onto the cellulose nanocrystal aerogel (AA-g-CNC) participated in hydrogen bonding and played a vital role in the swelling ability under various pH conditions. At low pH (<3.5), the negatively charged functional groups in AA-g-CNC combined with H+ reduced the adsorption capacity. As the pH increased, AA-g-CNC was deprotonated, which attracted positively charged metal ions and improved metal ion interactions through electrostatic interactions.

The solution temperature mainly affects the broadening nature of aerogel adsorbents and the solid/liquid interaction. With temperature, thermodynamic parameters are used to determine the nature of the adsorption process, i.e., exothermic or endothermic, spontaneity and randomness, and also to determine whether the temperature is favorable to the process or not [81]. Determining enthalpy, entropy, and Gibbs free energy is critical to understanding the amount of heat absorbed or released, the energy supplied by the system, and the randomness at the solid–liquid interface when adsorption occurs. Positive enthalpy indicates the endothermic nature of the process, while positive Gibbs free energy refers to the non-spontaneity of the process. However, adsorption can be categorized into physisorption and chemisorption depending on the Gibbs free energy [82].

Thermodynamic tests carried out by Talari et al. [72] on the adsorption of methylene blue (MB) and p-nitrophenol (PNP) on carbon fiber aerogel derived from bacterial cellulose indicated different adsorption mechanisms. For MB, the enthalpy value was positive, indicating an endothermic adsorption reaction, and adsorption increased with temperature. Concerning PNP, the negative variation in enthalpy indicated an exothermic reaction, and adsorption increased at lower temperatures.

The adsorption of oily contaminants is controlled by viscosity. Temperature affects the oil’s viscosity and, consequently, the oil adsorption capacity in aerogel [69]. Rafieian et al. [69] tested different temperatures (25, 40, and 60 °C). They confirmed that increasing the temperature reduces the oil’s viscosity (both engine and kitchen), causing easier and faster diffusion of the oil into the network of porous aerogel. However, the sharp reduction in oil viscosity (60 °C) led to poor oil adhesion to the pore walls and, consequently, greater oil drainage. Therefore, the highest oil adsorption capacity was achieved between 25 and 40 °C.

Contact time is another parameter that significantly affects the adsorption process. In general, adsorption by a porous solid tends to occur in three stages: (1) adsorption on the external surface, (2) gradual adsorption with intraparticle diffusion, and (3) interior surface adsorption and binding to active sites [83]. In order to explain the dynamics of the adsorption rate, pseudo-first-order and pseudo-second-order models are used. The pseudo-first-order model assumes that the adsorption rate in function to time is proportional to the difference between the adsorption capacity at equilibrium and the amount adsorbed at each time. The pseudo-second-order model describes the adsorption process considering chemisorption [84].

Liu et al. [70] observed a rapid increase in the adsorption capacity of heavy metal ions (Pb(II)(II), Cd(II)(II), and Cu(II)(II)) at the beginning of the process, followed by a decrease. This phenomenon is attributed to the initial adsorption phase, in which an empty surface has available adsorption sites. As the contact time between the aerogel and metals passes, heavy metal ions gradually fill the available free surface through the strong attraction between metal ions and carboxylate groups in the aerogel. The correlation coefficients calculated for kinetic models indicated that the adsorption kinetics of metal ions follows the pseudo-second-order model. These results suggest that the general adsorption tendency of metal ions is predominantly controlled by chemical adsorption.

The evaluation of the adsorption equilibrium is one of the main parameters considered in analyzing adsorbent effectiveness. Over time, several models have been employed to explain the adsorption equilibrium, including two- or three-parameter isothermal models [81]. Isotherm models are typically used to characterize the adsorption process, as they indicate how much of the adsorbate is being adsorbed by the adsorbent at a constant temperature. In this sense, a statistical study of isotherm models was carried out to identify the models that best fit and describe the most analyzed adsorption processes of pollutants in wastewater by different categories of nano adsorbents [82]. The most used isotherms are Langmuir and Freundlich. The Langmuir isotherm describes physisorption, and adsorption occurs in a homogeneous monolayer [85]. The Freundlich model assumes that adsorption occurs on a heterogeneous surface through multilayer sorption [86].

Talari et al. [72] noted that the equilibrium data for methylene blue (MB) follow the pattern of the Langmuir model, indicating monolayer adsorption on the homogeneous surface, with uniform adsorption energy and without reactions between the adsorbent molecules. In contrast, p-nitrophenol (PNP) adsorption fitted the Freundlich model, representing multilayer adsorption with the interaction between the adsorbed molecules and heterogeneity in the energy distribution at the active sites. In another work, Ma et al. [87] incorporated attapulgite nanorods (ATP) into a carboxylated nanocellulose matrix, a structure obtained through freeze-drying. Evaluating the adsorption of Cu(II) ions, the adsorption equilibrium data followed the pattern of the Langmuir model, with a maximum adsorption capacity of 116.5 mg/g. In this case, three main items were listed as major contributors to a high adsorption performance, these being the hierarchical porous structure, the good distribution and interaction between cellulose nanofibers and ATP, and the abundant presence of carboxyl groups (related to carboxylated cellulose).

### 4.2. Regeneration of Nanocellulose Aerogel

Regeneration is essential after adsorption to enable reuse, providing economic benefits. Regeneration aims to evaluate the possibility of desorption of the adsorbent. Several regenerative methods include adsorption/desorption cycles, thermal regeneration, precipitate formation, and others [82].

Cui et al. [52] conducted desorption using 40% (*w*/*w*) ethanol after adsorption of enrofloxacin. The hydrogen bond between the hydroxyl and enrofloxacin molecules in ethanol was stronger than that between the adsorbent surface. After elution, the adsorbent was lyophilized, and studies on reuse were carried out. After the fifth cycle, the removal efficiency of enrofloxacin decreased from 91.10 ± 1.16 to 75.71 ± 1.24%, showing good adsorption performance. After the sixth cycle, the removal efficiency decreased to 53.66 ± 1.48%. This reduction occurred due to the decrease in a load of ZIF-8 (zinc nitrate hexahydrate) on the surface of the aerogel after repeated elution, resulting in a decrease in active adsorption sites. The authors generally conclude that aerogel is an adsorbent produced from economical and recyclable material.

Mo et al. [35] proceeded with the desorption of heavy metal ions (Pb(II)(II), Cu(II)(II), Zn(II)(II), Cd(II)(II), and Mn(II)(II)) using a 1 mol/L EDTA solution, stirred at 25 °C for 3 h. A high % regeneration efficiency of 90% was observed over five consecutive adsorption/desorption cycles of Pb(II) ions. These results highlight excellent recyclability without compromising the adsorption performance of the aerogel.

## 5. Conclusions

Cellulose stands out as an excellent component in forming three-dimensional and porous aerogels owing to the organization of its structures through hydrogen bonds between the numerous hydroxyls present in its composition. Notwithstanding, in the context of the adsorption of environmental contaminants, cellulose aerogels have limitations due to their hydrophilicity in aqueous media attributed to these hydroxyls. Given this, researchers are committed to exploring methods of modifying the cellulose structure, using these hydroxyls as bridges to establish connections with other relevant components for the adsorption of different environmental contaminants.

The process of producing an aerogel involves the dispersion of cellulose, followed by gelation using chemical or physical crosslinkers, freezing (using a freezer or liquid nitrogen), and finally, drying, which can be carried out by supercritical CO_2_ or freeze-drying. Modification of cellulose aerogel can occur during the production process, incorporating other components, such as additional polymers, reinforcing nanomaterials, metallic particles, and organic components. This incorporation forms stable composites with diverse functional groups and active sites for the adsorption of various environmental contaminants. Alternatively, modification can occur after the formation of the aerogel using methods such as impregnation, coating, chemical vapor deposition, hydrothermal treatment, and carbonization with different organic compounds. These approaches aim to make the aerogel hydrophobic for the selective adsorption of organic contaminants.

The modifications implemented in the cellulose aerogel demonstrate excellent adsorption capacity for various types of environmental contaminants. However, the scarcity of research addressing the simultaneous adsorption of contaminants, simulating real effluents, and exploring real-scale applications is still evident. Furthermore, it is crucial to carry out studies covering economic viability, and investigations into more sustainable and ecological practices, as well as minimizing energy consumption to produce cellulose aerogel for the adsorption of contaminants. These analyses are essential to drive industries’ and consumers’ effective use of aerogel.

## Figures and Tables

**Table 1:** Modified nanocellulose aerogels and their adsorption capacity for environmental contaminants.

Cellulose Component	Methods	Modification	Environmental Contaminants	Adsorption Capacity	Authors
Cellulose nanofibrils (1%, 2%, and 3% *w*/*w*)	Crosslinking with epichlorohydrin (EPI) and freeze-drying	Crosslinking with beta-cyclodextrin	Phenol	148 μmol/g	[42]
Cotton nanocellulose (0.4% *w*/*w*)	Freezing in liquid nitrogen and freeze-drying	Crosslinking with sodium dodecyl sulfate (0.2% *w*/*w*)	Cyclohexane, ethyl acetate vacuum pump oil	206.79 g/g, 194.75 g/g 145.20 g/g	[43]
Bleached cellulose nanofibrils	Crosslinking with epichlorohydrin (EPI), freezing, and freeze-drying	Crosslinking with beta-cyclodextrin	Cyanotoxin (microcystin LR) methylene blue	0.078 mg/g 3.46 mg/g	[46]
Cellulose nanofiber oxidized by TEMPO (0.6% *w*/*w*)	Freezing in liquid nitrogen and freeze-drying	Crosslinking with graphene oxide (GO) (5% by weight) and trimethylolpropanotris-(2-methyl-aziridine) propionate—TMPTAP (1%, 3%, and 5% by weight of GO in relation to TMPTAP)	Pb^(II)^, Cu^(II)^, Zn^(II)^, Cd^(II)^ Mn^(II)^	571 mg/g, 462 mg/g, 361 mg/g, 263 mg/g 208 mg/g	[36]
Cellulose nanocrystals (1:1; 1:2, and 2:1)	Crosslinking with glutaraldehyde, freezing, and freeze-drying	Hydrophobic modification with tannic acid and functionalization with chitosan (3% *w*/*v*)	Sildenafil; basic blue dye 26; Cetylpyridinium chloride surfactant	86 mg/g, 375 mg/g, 390 mg/g.	[47]
Cattail Cellulose (*Typha orientalis*) (4% *w*/*w*)	Crosslinking with N, N′-methylenebisacrylamide (MBA), freezing, and freeze-drying	In situ growth of ZIF-8 (zinc nitrate hexahydrate) and immersion in 2-methylimidazole solution	Enrofloxacin	172.09 mg/g	[48]
Carboxylated cellulose nanofibers (12% *w*/*w*)	Freezing and freeze-drying	In situ crosslinking with Iron(III) chloride and aniline and polymerization induced by 1M sulfuric acid	Acid red G and methyl blue	600.7 mg/g, 1369.6 mg/g	[49]
Rice straw cellulose nanofibers (0.1 to 2% *w*/*w*)	Crosslinking with Epichlorohydrin (PAE), freezing in liquid nitrogen, and freeze-drying	Chemical vapor deposition (75 °C for 12 h) for hydrophobic coating with methyltrimethoxysilane	Hexane, acetone, toluene, dimethylformamide, chloroform, crude oil, pump and engine oils	From 98 g/g to 170 g/g	[51]
Cellulose fibers from corn agricultural waste (0.5 g for 20 mL of deionized water)	Freezing in liquid nitrogen and freeze-drying	Coating with 0.1 M ferrous sulfate	Tellurium	119.32 mg/g	[35]
Microcrystalline cellulose	Freezing and drying with supercritical CO_2_ and/or freeze-drying	Immersion in methanol coagulation bath	Kerosene	12 g/g	[55]
Cellulose nanofibers (0.5 wt%)	Crosslinking with polyvinyl alcohol (PVA), freezing, and freeze-drying	Dip-coating with fatty acids	Crude oil	60 g/g	[59]
Cellulose nanofibers (0.6, 0.9, and 1.2 wt%)	Freezing in liquid nitrogen and freeze-drying	Steam modification with hexadecyltrimethoxysilane (HDTMS)	Engine and cooking oil	78.8 g/g and 162.4 g/g	[60]
Cellulose nanocrystals	Freezing and freeze-drying	Hydrothermal treatment with acrylic acid	Pb^(II)^; Cd^(II)^; Cu^(II)^	1.026 mg/g, 898.9 mg/g and 872.4 mg/g	[61]
Cellulose nanofibers (0.05 wt%)	Freezing and freeze-drying	Crosslinking with nanochitosan (0.1 g), L-ascorbic acid (0.2 g), and graphene oxide (3.47 mL), and hydrothermal treatment (150 °C for 1 h)	Mineral oil, sesame oil, acetone, ethyl acetate, thiophene, pump oil, waste pump oil, kerosene ethyl alcohol,	171.85, 159.64, 153.22, 149.60, 139.93, 132.47, 176.82, 128.7, and 120.34 g/g	[62]
Bacterial cellulose	Freezing in liquid nitrogen and freeze-drying	Carbonization at 800 °C for 90min	Methylene blue and p-nitrophenol	106 mg/g and 60 mg/g	[63]
Microcrystalline cellulose	Freeze-drying	Crosslinking with ovalbumin and hydrothermal carbonization treatment (180 °C for 5.5 h)	Cr (VI) and Pb (II)	68 mg/g and 240 mg/g	[64]
